# Rater Dependence in Emergency Department Catheter-Appropriateness Audits: A Prospective Three-Rater Study of Item-Level Reliability

**DOI:** 10.3390/jcm15197574

**Published:** 2026-09-29

**Authors:** Ömer Çetin, Faruk Danış

**Affiliations:** 1 Department of Emergency Medicine, Faculty of Medicine, Bolu Abant İzzet Baysal University, 14030 Bolu, Türkiye; omrctinn@gmail.com; 2 Department of Emergency Medicine, Bolu İzzet Baysal Training and Research Hospital, 14030 Bolu, Türkiye

**Keywords:** urinary catheterization, guideline adherence, observer variation, quality indicators, health care, patient safety, clinical audit, reproducibility of results, catheter-associated urinary tract infections

## Abstract

**Background/Objectives**: Catheter-appropriateness audits report a single rate without a measure of its reliability. We measured how far an emergency department (ED) estimate depends on the clinician applying the checklist, and which items carry that dependence. **Methods**: We enrolled 117 consecutive adults catheterized in a tertiary ED between 21 January and 21 May 2026. Three emergency physicians independently classified every patient against a ten-item checklist drawn from the 2009 CDC/HICPAC guideline and the Ann Arbor criteria. A designated senior rater served as comparator, a clinical judgment rather than a criterion standard. We computed standalone rates, per-item Fleiss’ κ and Gwet’s AC1, patient-level agreement, and a post hoc counterfactual item-replacement analysis. **Results**: Ten patients (8.5%, 95% CI 4.7–15.0) had no valid indication according to the senior rater. Auditing alone, the two index raters would have reported 0.9% and 25.6%. Pooled agreement over 1170 patient-by-indication decisions was substantial by κ (0.630 and 0.707) and almost perfect by AC1 (0.885 and 0.925); excluding the complementary item changed neither. Patient-level agreement was lower (three-rater κ 0.199, AC1 0.792). Disagreement concentrated in hourly urine-output monitoring (AC1 0.617) and neurogenic bladder, endorsed in 44 patients by one rater and none by another (AC1 0.668). That item accounted for 48% of one rater’s excess endorsements; replacing that rater’s item-5 ratings with the senior rater’s narrowed the between-rater range from 30-fold to 6-fold. **Conclusions**: In this cohort the audit rate was rater-dependent, and the dependence concentrated in identifiable checklist items. Item-level operational definitions and prospective calibration should be evaluated as strategies for improving audit reliability, and per-item agreement should be reported with a prevalence-robust coefficient alongside κ.

## 1. Introduction

Indwelling urinary catheters are among the most frequently placed devices in acute care, and catheter-associated urinary tract infection remains one of the most common healthcare-associated infections [1,2]. Because infection risk accumulates with every catheter-day, the most effective preventive measure is not to place a catheter that is not clinically indicated [3]. The 2009 guideline of the Centers for Disease Control and Prevention and the Healthcare Infection Control Practices Advisory Committee (CDC/HICPAC) therefore defines a restricted set of appropriate indications for indwelling urethral catheters and recommends against use outside them [3]. Professional bodies define indications for emergency interventions in the same way, by enumerating the circumstances in which a procedure is warranted; what such documents rarely specify is how each criterion is to be operationalized at the bedside [4].

The emergency department (ED) is a decisive point in this pathway. A large share of the catheters that accompany a patient through admission are first placed during the ED encounter [5,6]. ED catheterization also falls outside guideline indications more often than catheterization on medical or surgical wards. A national analysis of United States EDs found that a substantial proportion of catheters placed in admitted patients were potentially avoidable [5]. A recent multicenter audit reported inappropriate use of 20.3% overall, but 35.1% in the ED specifically [7]. Several stewardship interventions have targeted this gap. Written indication criteria, physician champions, and structured de-implementation programs have reduced unnecessary use in several settings [8,9,10]. Infection-control interventions tracked over six years were likewise associated with a lower catheter-associated infection rate in a tertiary hospital [11].

Each of these interventions relies on the same instrument for monitoring: a periodic audit in which a clinician checks catheterized patients against an indication checklist and reports the proportion judged inappropriate. That proportion then serves as a quality indicator, compared across units and over time, and cited as proof an intervention worked. Observer dependence is a known limitation elsewhere in emergency care. Bedside ultrasonography for necrotizing soft-tissue infection, for instance, performs reasonably but stays user-dependent [12], and operator dependence is a recognized source of error across emergency ultrasonography as a whole [13]. For appropriateness audits it is rarely raised. Yet if two equally qualified clinicians reviewing identical records would reach materially different rates, the indicator measures the auditor almost as much as the practice.

The reliability of appropriateness judgments has, in fact, been examined before. Shekelle and colleagues showed that the RAND/UCLA appropriateness method reproduces poorly at the level of the expert panel: two panels of equal standing, rating the same indications, disagreed enough to move the measured rate of inappropriate use by a factor of more than two [14]. Review criteria developed by such panels require field testing before they can be treated as valid and reliable measures of care [15]. Inter-rater agreement in routine medical-record auditing has also been evaluated prospectively, and was found to improve when raters meet to reconcile their criteria [16]. In each case, what varied was the panel that wrote the criteria, or the audit process as a whole. A complementary question remains unquantified, and it is the one that matters to a department reporting a rate: with the criterion set held fixed and the records held fixed, how much does the published number depend on which clinician applies it, and which individual criteria carry that dependence? Emergency medicine answers this question for its decision rules before adopting them, through prospective validation in the population of use [17]; the field’s methodological standards require it [18]. Catheter-appropriateness checklists have not been held to the same standard.

The statistics ordinarily used to answer such a question are themselves vulnerable here. Agreement is conventionally summarized with Cohen’s or Fleiss’ κ. Both behave paradoxically when one category is rare. With skewed marginal distributions, κ can fall to low or even zero values despite very high observed agreement, because the expected-agreement term is inflated [19,20]. Inappropriate catheterization is, by design, the rare category in a well-run department. Prevalence-robust coefficients such as Gwet’s first-order agreement coefficient (AC1) were developed for this situation. They recover reliability more faithfully under skew [21,22]. We therefore conducted a prospective cohort study in which every catheterized patient was classified independently by three emergency medicine specialists against a checklist derived from the CDC/HICPAC indications. Our objectives were several. First, we aimed to determine the appropriateness of ED bladder catheterization according to a designated senior rater; second, to quantify how far the audit result would change had each rater performed the audit alone; third, to locate which checklist items carry the disagreement, and to quantify how much of the between-rater spread is attributable to individual items; and finally, we sought to characterize early microbiological findings after catheterization.

## 2. Materials and Methods

### 2.1. Study Design and Setting

We conducted a single-center, non-interventional, prospective observational cohort study with a nested inter-rater reliability analysis in the adult ED of a tertiary referral hospital in Türkiye. Patients were enrolled consecutively between 21 January 2026 and 21 May 2026. The investigators observed routine practice and did not influence any catheterization decision; the decision to place a catheter and the choice of device were made entirely by the treating ED physicians. The study is reported in accordance with the Strengthening the Reporting of Observational Studies in Epidemiology (STROBE) statement [23]. The data for this study were obtained from the same patient cohort as a residency (medical specialty) thesis completed at the Department of Emergency Medicine, Faculty of Medicine, Bolu Abant İzzet Baysal University.

### 2.2. Participants

Consecutive adults aged 18 years or older who underwent bladder (urethral) catheterization in the ED and whose catheter remained in situ for at least 24 h were eligible. Written informed consent for participation was obtained from each participant or a legal representative before enrollment. Patients with a pre-existing diagnosis of urinary tract infection, patients whose catheter was placed by another unit or before ED arrival, and patients whose records were inaccessible or incomplete did not meet the eligibility criteria and were not enrolled. Enrollment ran continuously over the four months and did not depend on the investigators’ shifts. Every patient who met the eligibility criteria during the study period was enrolled, in order of presentation. No screening log was kept. The number of catheterized patients assessed against the eligibility criteria is therefore not available, and a consecutiveness index of the kind proposed for quality-improvement audits [24] cannot be calculated. All 117 enrolled patients were analyzed; there were no post-enrollment exclusions and no loss to follow-up.

### 2.3. Indication Assessment and the Senior-Rater Comparator

Appropriateness was defined against a ten-item checklist of indications for indwelling urethral catheterization. Items 1 to 4, 6 and 7 were taken from the appropriate-indications table of the 2009 CDC/HICPAC guideline [3]. Three items were added from the Ann Arbor criteria for appropriate urinary catheter use, which were developed by the RAND/UCLA method for hospitalized medical patients [25]: neurogenic bladder dysfunction, non-traumatic gross hematuria, and exchange of a chronic indwelling catheter. The checklist comprised: (1) acute urinary retention or bladder outlet obstruction; (2) accurate measurement of hourly urine output in critically ill patients; (3) perioperative use for selected procedures; (4) prolonged immobilization after potentially unstable spinal or pelvic injury; (5) neurogenic bladder dysfunction; (6) healing of open sacral or perineal wounds in incontinent patients; (7) end-of-life comfort care; (8) non-traumatic gross hematuria requiring monitoring or irrigation; (9) scheduled exchange of a chronic indwelling catheter; and (10) no valid indication, that is, inappropriate catheterization. Each item carried a one-line descriptor. The form, translated into English, is reproduced in the Supplementary Materials (Table S1). The descriptor for item 5 named acute spinal injury, stroke, multiple sclerosis and Guillain-Barré syndrome as example causes of voiding dysfunction. The form was supplied in writing. No calibration session, pilot rating or content-validation exercise preceded the audit, and the raters did not discuss any item with one another before or during rating. A patient was classified as appropriately catheterized if at least one of items 1–9 applied; item 10 is therefore the complement of items 1–9, and this relationship held exactly for all three raters in the data.

Three emergency medicine specialists rated every patient against all ten items, independently of one another and blinded to the treating physician’s rationale. Two served as index raters, with five and nine years of post-specialty experience at the time of rating (index rater 1 and index rater 2). The third was a senior specialist with thirteen years of post-specialty experience. Rating began after enrollment closed. For every patient, each rater received the same ED-period documentation: the ED physician notes and ED discharge summary, nursing observations, the orders and results generated during the ED stay, and the diagnoses recorded in the hospital information system before or during the visit. Information generated after admission, including the day-3 urinalysis and culture results, was not provided to any rater. Undocumented reasoning was therefore unavailable to all three raters alike. None could consult the treating team. The senior specialist’s classification was defined a priori as the comparator for the primary appropriateness outcome. We refer to it as the designated senior rater’s classification rather than as a reference standard. It is a clinical judgment made with the same checklist, and seniority does not establish criterion validity. It was made independently of both index raters for every patient, not derived from their agreement, so no part of it is a function of the index ratings. The senior specialist is not an author of this manuscript. One index rater is an author (F.D.). He took no part in the senior rater’s classification and was blinded to the other index rater’s assessments.

### 2.4. Variables and Outcomes

The primary outcome was appropriateness of ED bladder catheterization according to the designated senior rater. The principal reliability outcomes were the inappropriateness estimate each rater would have reported had that rater audited the cohort alone, agreement between raters at the level of the individual indication decision, and agreement on the patient-level verdict. Three levels of inference are kept distinct throughout: agreement among the three raters; agreement of each index rater with the designated senior rater; and criterion validity, which this design does not establish. Prespecified secondary outcomes were early microbiological and urinary findings on day 3 after catheterization: whether urinalysis was evaluated, whether a urine culture was obtained, whether the culture grew an organism, and the organism isolated. These were obtained prospectively from routine institutional clinical and laboratory records. In keeping with the guideline definition, a positive culture was recorded as an early microbiological finding and was not equated with a clinical diagnosis of catheter-associated urinary tract infection [3].

Independent variables included age, sex, presenting complaint, ED diagnosis, comorbidities, catheter type (French size and material), and indication category. Mechanical and traumatic complications of catheterization were not analyzed. The source records did not document these events in a structured form.

### 2.5. Sample Size

The sample size was fixed a priori for the reliability analysis using the method of Walter and colleagues [26]. Assuming a two-sided α of 0.05, 80% power, an acceptable agreement of κ = 0.60 and an expected agreement of κ = 0.80, the minimum requirement was 108 patients. We enrolled 117. Because each patient was assessed against ten indications, the design yielded 1170 matched patient-by-indication decisions and, across the two index raters, 2340 rater-level assessments. 

### 2.6. Statistical Analysis

Analyses were performed in IBM SPSS Statistics for Macintosh, version 29.0 (IBM Corp., Armonk, NY, USA) and in R version 4.6.1 (R Foundation for Statistical Computing, Vienna, Austria) within RStudio version 2026.09.0+174 (Posit Software, PBC, Boston, MA, USA), with the R packages irr, irrCAC, lme4 and boot [27]. Continuous variables are summarized as mean ± standard deviation and categorical variables as counts and percentages. Proportions are reported with 95% Wilson confidence intervals (CIs). A single reproducible script generated every reported value, and package versions were recorded with sessionInfo(). Agreement analyses are reported in line with the Guidelines for Reporting Reliability and Agreement Studies [28].

The analysis proceeded in five stages. First, for each rater we computed the proportion of patients that rater would have classified as inappropriate had that rater audited the cohort alone, along with the proportions obtained under unanimous and any-rater decision rules. Second, for each of the ten indications, we tallied the number of patients endorsed by each rater, the number endorsed unanimously, and the number contested; per-indication Fleiss’ κ and Gwet’s AC1 were also calculated. Third, we compared each index rater with the designated senior rater. Across the 1170 decisions we report observed agreement, Cohen’s κ, Gwet’s AC1, and the positive and negative agreement of Cicchetti and Feinstein [19], which are the proportions of decisions endorsed by both raters among those endorsed by either, and the corresponding proportion for non-endorsement. We also report, among the decisions the senior rater endorsed, the share the index rater endorsed as well. Because item 10 is the arithmetic complement of items 1 to 9, every pooled statistic was recomputed on items 1 to 9 alone (1053 decisions). Agreement on the patient-level verdict, appropriate or inappropriate, was analyzed separately, pairwise and across all three raters. Fleiss’ κ and a three-rater AC1 were computed across all three raters. κ values are described with the Landis and Koch labels [29]. We use these labels descriptively only: they were not developed for AC1, and the two coefficients define chance agreement differently [30]. CIs for the pooled coefficients were obtained by bootstrap resampling of patients (2000 replicates), which preserves the clustering of the ten decisions within each patient. Fourth, a generalized linear mixed model with a random intercept for patient identity tested whether age or sex was associated with an index rater departing from the senior rater’s classification across the 2340 rater-level assessments. Age was centered on its mean to aid convergence, and results are reported as odds ratios (ORs) with 95% Wald CIs.

Fifth, because the between-rater spread proved to be large, we ran a post hoc item-level analysis. Its aim was to establish whether that spread was distributed across the checklist or concentrated in particular items. For the item contributing most of one rater’s excess endorsements relative to the senior rater, we recomputed every rater’s patient-level inappropriateness estimate under two counterfactuals. In the first, that rater’s ratings on the item were replaced with the senior rater’s ratings (counterfactual item replacement). In the second, the item was removed from the checklist for all raters (item removal). Item 10 is the complement of items 1 to 9. An item’s influence on the headline rate is therefore arithmetically traceable in this way. By construction, item replacement moves the rater’s estimate toward the senior rater’s. It measures how much of the spread one item carries; it does not reproduce a calibration intervention. We specified the analysis after seeing the spread. It is reported as hypothesis-generating throughout.

Analyses of correlates of inappropriateness and of the microbiological findings were treated as exploratory and restricted to univariate tests: Welch’s t-test for age and Fisher’s exact test for categorical comparisons. Multivariable modeling of these outcomes was conditional on an adequate number of events per variable, a condition that, as reported below, was not met. Two-sided *p*-values below 0.05 were considered statistically significant.

### 2.7. Ethics

The study was conducted in accordance with the Declaration of Helsinki and approved by the Non-Interventional Clinical Research Ethics Committee of Bolu Abant İzzet Baysal University (meeting no. 43, decision no. 2026/10, 20 January 2026). Data collection began only after approval was granted. Written informed consent for participation was obtained from every participant or a legal representative before enrollment; the consent form was distributed to, and signed by, every participant or legal representative.

## 3. Results

### 3.1. Cohort Characteristics

**Figure 1:**
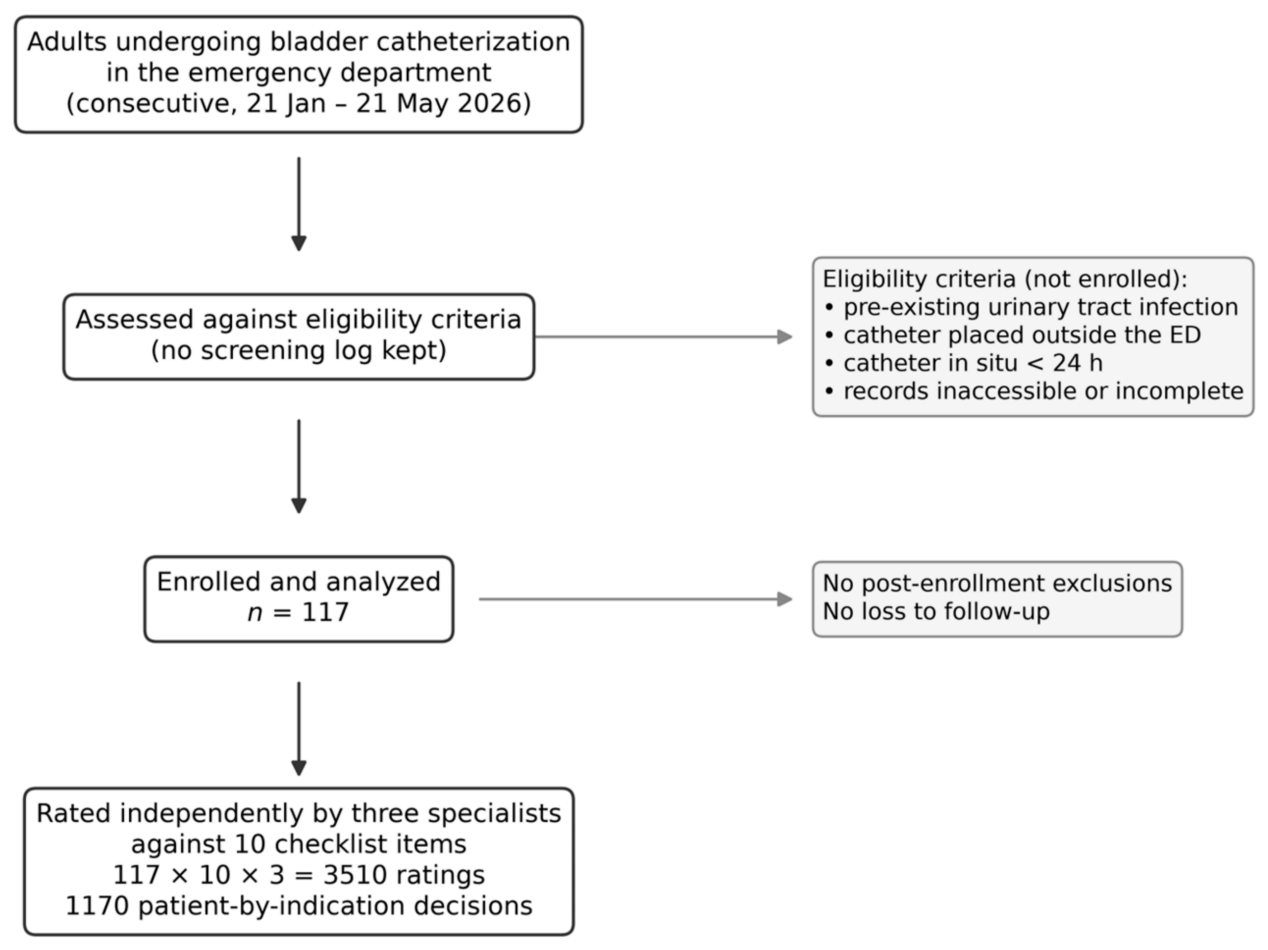
Participant Flow: Every adult who met the eligibility criteria during the study period was enrolled in order of presentation; no screening log was kept, so the number assessed against the criteria is not available. All 117 enrolled patients were rated independently by three emergency medicine specialists against all ten checklist items. ED, emergency department.

A total of 117 consecutive adults undergoing ED bladder catheterization were enrolled and analyzed (Figure 1). The mean age was 73.74 ± 13.11 years (range 21–96) and 62 patients (53.0%, 95% CI 44.0–61.8) were women. The cohort carried a heavy burden of chronic disease: hypertension in 71 patients (60.7%), diabetes mellitus in 39 (33.3%), coronary artery disease in 33 (28.2%), heart failure in 23 (19.7%), previous cerebrovascular accident in 14 (12.0%), chronic kidney disease in 9 (7.7%) and benign prostatic hyperplasia in 1 (0.9%). The commonest presenting complaints were dyspnea (29 patients, 24.8%), general deterioration (13, 11.1%) and abdominal pain (12, 10.3%); the commonest ED diagnoses were cerebrovascular accident (18, 15.4%), pneumonia (14, 12.0%) and acute kidney injury (11, 9.4%). Cohort characteristics are shown in Table 1.

Day-3 urinalysis was performed in 24 of 117 patients (20.5%) and a urine culture was obtained from 22 (18.8%), with growth in 10; sampling followed routine care rather than a surveillance protocol, so these counts describe a selected subgroup and not incidence (Table S5). We report them because exposure to catheter-days is the reason appropriateness is audited at all.

### 3.2. Appropriateness

#### According to the Designated Senior Rater

According to the designated senior rater, 107 patients (91.5%) had at least one valid indication and 10 (8.5%, 95% CI 4.7–15.0) had none and were classified as inappropriately catheterized. Thirteen patients met more than one valid indication. Accurate measurement of hourly urine output in critically ill patients was by far the most common indication, recorded in 74 patients (63.2%), followed by perioperative use (26, 22.2%), prolonged immobilization after trauma (8, 6.8%), neurogenic bladder (6, 5.1%) and acute urinary retention (5, 4.3%). Non-traumatic gross hematuria and end-of-life comfort care accounted for two patients each (1.7%); no patient was catheterized for wound care in incontinence or for scheduled catheter exchange (Table S2).

### 3.3. The Audit Result Depends on Who Performs It

**Figure 2:**
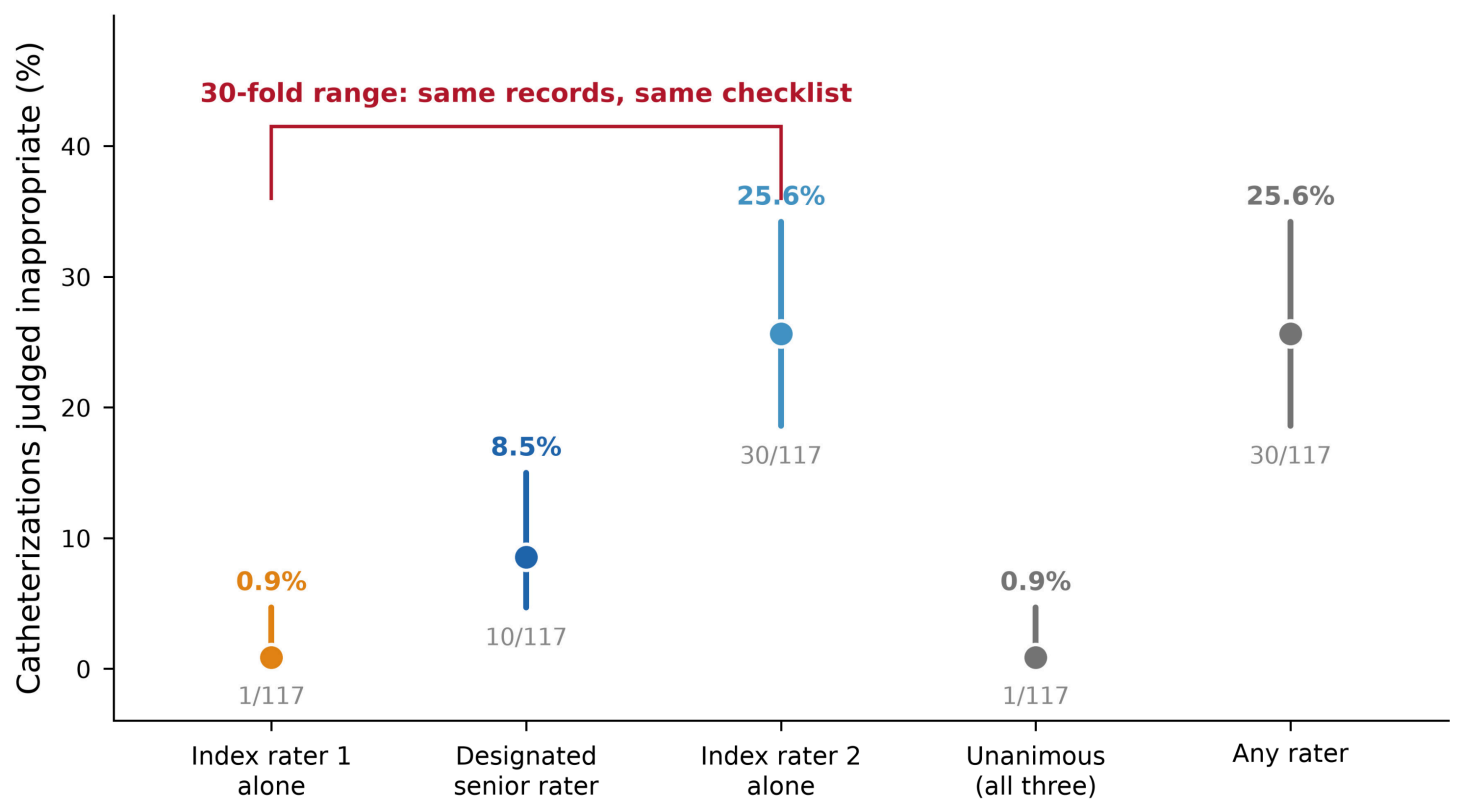
Inappropriate-catheterization estimates for the same 117 records under five decision rules. Points are point estimates and vertical lines 95% Wilson confidence intervals; counts below each point are the numerator and denominator. The two index raters bracket the designated senior rater on either side, spanning a 30-fold range. Colors identify the rater or decision rule: orange, index rater 1; dark blue, the designated senior rater; light blue, index rater 2; gray, the unanimous and any-rater composite rules. The red bracket marks the span between the two index raters.

The three specialists classified the same 117 records against the same checklist, yet the inappropriateness rate each would have reported differed markedly (Table 2, Figure 2). Index rater 1 would have identified 1 patient (0.9%, 95% CI 0.2–4.7) as inappropriately catheterized and index rater 2 would have identified 30 (25.6%, 95% CI 18.6–34.2), against 10 (8.5%) according to the senior rater, with a 30-fold range between the two extremes. Only 1 patient was judged inappropriate by all three raters, whereas 30 were judged inappropriate by at least one; the 29 patients between these bounds (24.8% of the cohort) constitute a contested zone in which the classification depends on which specialist performs the audit.

The two index raters diverged in opposite directions rather than sharing a common bias. Index rater 1 classified one patient as inappropriately catheterized. That patient was among the 10 the senior rater classified the same way; the other 9 were not identified. Index rater 2 classified 30 patients as inappropriately catheterized, including all 10 identified by the senior rater and 20 whom the senior rater judged to have a valid indication. One reading was narrower than the senior rater’s, the other broader, and together they bracket the senior rater’s estimate on either side. Agreement on this patient-level verdict is reported in Section 3.5.

### 3.4. Where the Disagreement Lies

**Figure 3:**
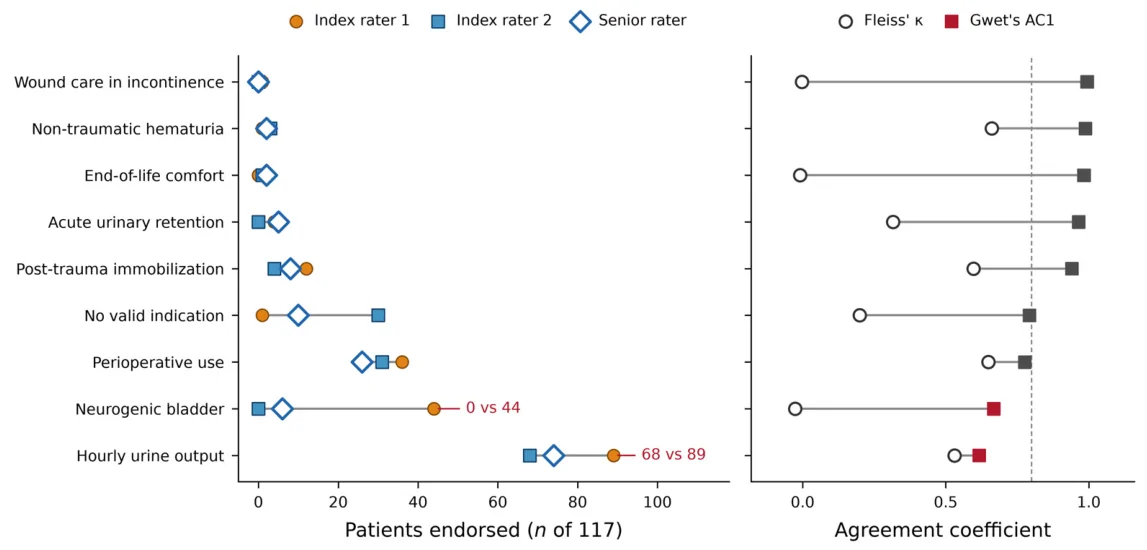
Item-level divergence and agreement across the nine ratable indications, ordered by Gwet’s AC1 (lowest at the bottom). Left: patients endorsed for each indication by each rater; the gray connecting line spans the three raters. Right: Fleiss’ κ (open circles) and Gwet’s AC1 (filled squares) for the same indication, with the two items carrying genuine disagreement in red and the remaining seven in gray; the dashed line marks AC1 = 0.80. Scheduled catheter exchange is omitted because no rater endorsed it, leaving κ undefined.

Disagreement was not distributed evenly across the indication set (Table 3, Figure 3). Two items accounted for most of the unevenness. They differed in kind. Accurate measurement of hourly urine output in critical illness, which justified 63.2% of all catheterizations and was the sole justification for 63 patients (53.8% of the cohort), had the lowest Gwet’s AC1 of all ten indications (0.617). Of the 96 patients in whom any rater endorsed it, only 59 were endorsed unanimously and 37 were contested. Index rater 1 endorsed this indication in 89 patients, the senior rater in 74 and index rater 2 in 68.

Neurogenic bladder dysfunction showed a different and more extreme pattern. The senior rater recorded it in 6 patients, and index rater 2 recorded 0, while index rater 1 endorsed it in 44. No patient was endorsed unanimously. A total of 44 cases were contested, more than the 37 contested for hourly urine output, yielding the second-lowest AC1 of the set (0.668). Hourly urine-output measurement requires a judgment about severity. Neurogenic bladder dysfunction, by contrast, is a documented diagnosis. A spread of 0 to 44 across the same records therefore reflects a difference in how the item itself was construed, rather than a difference in clinical threshold.

The two coefficients ordered the indications differently. By Fleiss’ κ, hourly urine-output measurement (κ = 0.531) ranked sixth among the nine ratable categories. It fell below neurogenic bladder (κ = −0.026), end-of-life comfort (−0.009), wound care in incontinence (−0.003), no valid indication (0.199), and acute urinary retention (0.316). These five are low-prevalence categories. In each, κ was depressed while the AC1 for the same five ranged from 0.668 to 0.994. Under the prevalence-robust coefficient, though, the ordering inverted: the two items carrying genuine disagreement, hourly urine output and neurogenic bladder, were ranked last and second-last.

In a generalized linear mixed model of departure from the senior rater’s classification across 2340 rater-level assessments, neither patient age nor sex explained the rater deviation (Table S4).

### 3.5. Pooled and Patient-Level Agreement, and the Divergence Between κ and AC1

Across the 1170 patient-by-indication decisions, both index raters agreed closely with the designated senior rater in absolute terms. Observed agreement was 91.2% for index rater 1 and 94.0% for index rater 2. Of the decisions the senior rater endorsed, index rater 1 also endorsed 81.95% and index rater 2 75.19%. Chance-corrected agreement was nonetheless only substantial: Cohen’s κ 0.630 (95% CI 0.561–0.697) and 0.707 (0.619–0.788), with Fleiss’ κ across all three raters 0.611 (0.551–0.668). Gwet’s AC1 for the same data was almost perfect at 0.885 (0.859–0.910) and 0.925 (0.904–0.945), with a three-rater AC1 of 0.886 (0.869–0.903). The κ-to-AC1 difference was 0.255, 0.218 and 0.275 respectively, moving the descriptive label from the substantial to the almost-perfect Landis and Koch band in every comparison (Table 4 and Table S3, Figure 3).

Item 10 is the complement of items 1 to 9, so we recomputed the pooled coefficients on items 1 to 9 alone (1053 decisions). The values barely moved. κ was 0.647 (0.580–0.710) and 0.756 (0.678–0.831), AC1 0.881 (0.856–0.905) and 0.941 (0.922–0.958), and three-rater Fleiss’ κ 0.652 (0.598–0.707) with AC1 0.897 (0.880–0.912). The complementary item therefore did not drive the pooled result (Table 4).

Agreement on the patient-level verdict was a different matter. Index rater 1 and the senior rater assigned the same verdict to 108 of 117 patients (92.3%), but positive agreement on the inappropriate verdict was 18.2%, giving κ 0.169 (0.000–0.487) and AC1 0.916 (0.852–0.965). Index rater 2 and the senior rater agreed on 97 patients (82.9%), with positive agreement 50.0%, κ 0.426 (0.239–0.603) and AC1 0.761 (0.640–0.861). The two index raters agreed with each other on 88 patients (75.2%), κ 0.049 (0.000–0.165) and AC1 0.678 (0.531–0.794). Across all three raters, Fleiss’ κ was 0.199 (0.060–0.337) and AC1 0.792 (0.703–0.864). This is not a contradiction. High decision-level agreement coexisted with low agreement on the verdict an audit actually reports, because the verdict turns on the few patients in whom every one of nine valid indications is denied.

### 3.6. One Checklist Item Carries Most of the Spread

Index rater 1 endorsed a valid indication (items 1–9) 187 times against 123 for the senior rater, an excess of 64 endorsements; index rater 2 endorsed 107. Of index rater 1’s 79 decisions in which a valid indication was endorsed where the senior rater recorded none, 38 (48%) fell on the single item of neurogenic bladder. In the 38 patients where that rater endorsed neurogenic bladder and the senior rater did not, the senior rater recorded hourly urine-output monitoring in 30, perioperative use in 9, post-trauma immobilization in 3, and no valid indication in 4. Of the nine patients the senior rater classified as inappropriately catheterized and index rater 1 did not, that rater had endorsed neurogenic bladder in four, and in three it was the sole endorsement.

**Figure 4:**
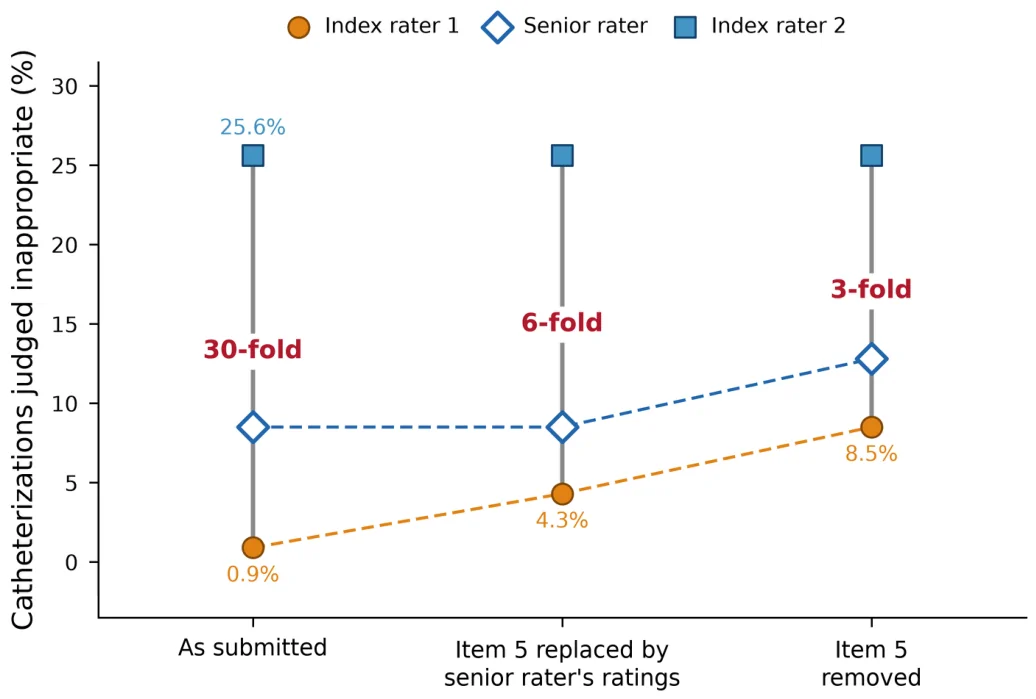
Post hoc counterfactual item-replacement analysis. Inappropriate-catheterization estimates for each rater as submitted, after replacing index rater 1’s ratings on checklist item 5 (neurogenic bladder dysfunction) with the senior rater’s ratings, and after removing item 5 from the checklist for all raters. Gray lines span the two index raters; the dashed lines connect the same rater across the three scenarios, orange for index rater 1 and blue for the designated senior rater, while index rater 2’s estimate is unchanged and is therefore not connected; the annotation gives the between-rater range in each scenario. This analysis is post hoc.

The post hoc item-level analysis quantified the consequence (Table 5, Figure 4). As submitted, the two index raters bracketed the senior rater at 0.9% and 25.6%, a 30-fold range. Replacing index rater 1’s ratings on item 5 with the senior rater’s ratings, and leaving every other item untouched, raised that rater’s estimate from 0.9% to 4.3% (5/117) and narrowed the range to 6-fold. Removing item 5 from the checklist for all raters brought index rater 1 to 8.5% (10/117) and the senior rater to 12.8% (15/117). This narrowed the range further, to 3-fold. The headline spread is therefore not a diffuse property of the instrument. Most of it traces back to how one item was applied by one rater. Because replacement substitutes the senior rater’s own ratings, it necessarily moves the estimate toward the senior rater’s; it shows how much of the spread the item carries, not what a training intervention would achieve. This analysis is post hoc and hypothesis-generating.

## 4. Discussion

### 4.1. Principal Findings

Three emergency medicine specialists applied one checklist to one set of 117 records and would have reported inappropriate catheterization in 0.9%, 8.5% and 25.6% of the cohort. The audit result was therefore rater-dependent across a 30-fold range, and the two index raters departed from the senior rater in opposite directions: one identified 1 of the senior rater’s 10 inappropriate cases and no others, the other identified all 10 together with 20 more. Our second finding constrains the first and is the more useful of the two. The disagreement was not spread across the instrument. It concentrated in two of ten items, and for one of them, neurogenic bladder dysfunction, 48% of a single rater’s excess endorsements were attributable to that item alone. Replacing that one item’s ratings narrowed the between-rater range from 30-fold to 6-fold, and removing it narrowed the range to 3-fold. On these data, the instability of a checklist-based appropriateness rate was localizable. Whether it is also remediable is a separate question. The data raise this question. They do not answer it.

### 4.2. Relation to Previous Work

Others have shown before that appropriateness judgments reproduce imperfectly, but at a different level of the measurement chain. Shekelle and colleagues convened two expert panels of equal standing to rate the same indications by the RAND/UCLA method and found that the panel itself moved the measured rate of inappropriate use by more than a factor of two [14]. The review criteria produced by such panels need field testing before they can be treated as valid quality measures [15]. Prospective evaluation of routine record auditing has also shown that inter-rater agreement is not fixed; it improves when raters meet to reconcile criteria [16]. Those studies varied who wrote the criteria, or how the audit program was run. We held the criterion set and the records fixed and varied only the clinician applying them, which isolates the component of variance a department actually controls when it commissions an audit, and we resolved that variance to the level of the individual checklist item. The distinction matters practically: a department cannot rewrite the CDC/HICPAC indication set, but it can calibrate its auditors on the two items that carry the disagreement. The same instability appears when the criterion set changes rather than the panel. Applying the Chinese, AGS/Beers and STOPP criteria to 131,894 outpatient prescriptions produced inappropriate-prescribing prevalences of 29.00%, 27.77% and 19.85%, with only moderate concordance between sets [31]. There the instrument moved the rate. Here, the instrument was fixed and the rater moved it. The mechanism appears to be the same. The pattern is also not confined to audits. When two cardiologists classified cardiovascular risk by clinical impression and the result was set against a standardized calculator, agreement was poor (κ 0.208), and the classification a patient received changed the lipid-lowering treatment and the target attainment recorded for that patient [32]. Clinician-dependent classification, in other words, can alter the downstream estimate of care quality, which is exactly what a rater-dependent appropriateness rate does.

Observer dependence is well recognized for other assessment tools in emergency care. Bedside ultrasonography contributes usefully to the recognition of necrotizing soft-tissue infection, yet it remains user-dependent. Its performance is therefore reported alongside this caveat rather than as a fixed operating characteristic [12]. Operator-related error is among the principal sources of error cataloged for emergency ultrasonography generally [13]. Emergency medicine also has an established convention for instruments that generate a binary verdict. Such tools are validated prospectively in the population of intended use before adoption, as the methodological standards for decision rules require [18] and as we and others have done for a decision rule governing head computed tomography after ground-level falls in older adults [17]. Appropriateness checklists produce exactly this kind of binary verdict. They are used to judge institutional performance, yet they have not been held to the same standard. Our results indicate why they should be. The pattern reaches beyond clinical measurement. Six raters applying a structured risk-of-bias instrument to prediction studies reached only slight to moderate agreement, with multi-rater AC1 ranging from 0.071 to 0.535 across domains, and after customized training the range rose to 0.294 to 0.780 [33]. Agreement there was domain-dependent and responsive to training. The question is being put to other structured clinical instruments as well. A systematic review and meta-analysis of CT-based subarachnoid hemorrhage grading scales pooled Cohen’s κ and the intra-class correlation across twenty reliability studies, using a dedicated appraisal tool for that literature [34]. Instrument reliability is treated there as a primary research question rather than a methodological footnote.

Our senior-rater estimate of 8.5% inappropriate use sits at the low end of the published range. National United States data identified a substantial share of ED-placed catheters as potentially avoidable [5]. A recent multicenter audit reported 35.1% in the ED specifically [7]. Appropriateness has also been shown to change dynamically during a hospital stay, rather than being fixed at the moment of insertion [35]. The same cohort would be reported at 0.9% or 25.6% depending on the auditor. This suggests that part of the heterogeneity across published audits reflects the auditors rather than the practices being audited. None of this invalidates the literature. It does, however, argue against reading small differences between institutions or between time points as differences in care.

### 4.3. Why These Two Items Disagreed, and What It Implies

The two items that carried the disagreement failed for different reasons, and the distinction determines the remedy. Accurate measurement of hourly urine output in critically ill patients justified 63.2% of catheterizations here and was the sole justification for 53.8% of the cohort. The CDC/HICPAC wording specifies neither what constitutes critical illness nor how frequently output must be measured to require an indwelling catheter [3], and our descriptor, which listed shock, sepsis, polytrauma and the need for intensive-care follow-up as examples, still left the severity threshold to the rater. Clinicians therefore supply their own thresholds, and reasonable clinicians supply different ones; the resulting AC1 of 0.617 is what threshold ambiguity looks like in a reliability statistic. This pattern is familiar in emergency medicine. The diagnostic performance of a measurement is determined by the cut-off chosen for it, whether that measurement is an end-tidal carbon dioxide value integrated into a termination-of-resuscitation rule [36] or an optic nerve sheath diameter used to identify hypertensive emergency [37]. A cut-off selected on the data at hand is also known to inflate apparent performance [38]. In each case the instrument becomes usable only once the threshold is stated explicitly. The same applies here. The remedy for item 2 is an operational definition, a stated severity criterion and a stated measurement interval, not exhortation to greater care.

Neurogenic bladder dysfunction is a different problem. It is a documented diagnosis rather than a severity judgment, so a spread from 0 to 44 patients across identical records cannot be explained by threshold ambiguity. The form itself supplies the most plausible explanation. Its descriptor for item 5 named stroke among the example causes of voiding dysfunction, and cerebrovascular accident was the commonest ED diagnosis in this cohort. One rater read the item as satisfied by a qualifying neurological diagnosis; the senior rater and the other index rater required documented voiding dysfunction attributable to it. Both readings are defensible against the wording as written, which suggests that insufficiently specific item wording contributed substantially to the observed disagreement; with only three raters, the design cannot fully separate item ambiguity from an idiosyncratic reading by a single rater. The remedy this points to differs from that for item 2: explicit inclusion and exclusion wording that separates the diagnosis from the dysfunction, and a calibration exercise on shared cases before the audit begins. The same rater’s narrow reading of the explicit inappropriate-use item and broad reading of this valid-indication item are arithmetically linked. Item 10 is the complement of items 1–9, so over-endorsing a valid indication mechanically suppresses the inappropriateness count. An audit that reports only the headline rate cannot distinguish a department with good practice from an auditor with a broad reading of one item.

The choice of agreement coefficient changed not only the magnitude of estimated reliability but which items would have drawn attention. Chance-corrected κ placed hourly urine-output measurement (κ = 0.531) below five low-prevalence items whose AC1 values ranged from 0.668 to 0.994, and assigned neurogenic bladder a κ of −0.026, which is indistinguishable from the values for items on which the raters simply never disagreed because almost no patient qualified. An analyst reading κ alone would look first at categories where little was at stake, and away from the item that was moving the headline rate. The two coefficients are not competing estimates of one quantity. They define chance agreement differently and answer related but distinct questions [30,39], and under markedly imbalanced marginals they diverge for that reason [19,20]. Prevalence-robust coefficients such as Gwet’s AC1 were designed for this marginal structure [21] and perform closer to true reliability in simulation [22,40]. Methodologists recommend them alongside κ rather than instead of it [41], and prospective record-audit programs have adopted them for this reason [16]. Our data support reporting both, per item, and reading the pair rather than either alone: the 0.255 to 0.275 gap between the two coefficients is itself the signal that a rare-category structure is present. The Landis and Koch labels should be read with the same caution. They were proposed for κ and carry no inferential weight, and the same numerical value does not mean the same thing for the two coefficients [29,30].

There is a broader point about candidate quality indicators. A plausible measurement is not automatically a valid one, and the literature on quality indicator methods asks that an indicator’s reliability be demonstrated before it is used to compare providers [42]. In a previous cohort of cardiac arrest patients, we found that of two physiologically reasonable monitoring parameters, one discriminated outcome and the other did not [43]. A single-rater appropriateness rate is plausible in the same way. On these data, though, it fails to meet the reliability requirement its use presupposes. This does not mean abandoning appropriateness auditing. The practice gap it targets is real [5,7]. Rather, the rate should be reported alongside the reliability evidence that would be demanded of any other measurement instrument.

### 4.4. Implications for Practice

Three consequences follow for departments that audit catheter appropriateness. First, a rate produced by one reviewer should not be treated as a stable performance measure, and differences between units or between audit cycles should not be interpreted as changes in care unless the reviewers were the same or were demonstrably calibrated. Second, calibration should be item-level and should precede the audit. Our counterfactual analysis indicates that most of the spread in this audit sat in the reading of two items, and agreeing their operational reading in advance is therefore the strategy most worth testing. Third, audit reports should include per-item agreement with both a chance-corrected and a prevalence-robust coefficient, so that the items driving instability are visible to whoever acts on the report. These are modest additions to an existing workflow. They are also the same additions the field already requires of a prognostic or diagnostic measure before it enters institutional decision-making [44], and the validation steps expected of a prediction model before clinical use follow the same logic [45]. Departments should also expect the measurement itself to need maintenance. A Lean-driven improvement initiative in an ED found that a simple pain scale, available for repeated use throughout a patient’s stay, was still monitored less consistently than blood pressure or oxygen saturation [46]. An instrument that is easy to apply is not therefore applied uniformly.

### 4.5. Strengths and Limitations

The principal strengths are the prospective consecutive design, the fully crossed rating structure in which all three specialists classified all 117 patients against all ten items, yielding 1170 matched decisions, and a senior-rater comparator that was determined independently of both index raters for every patient rather than derived from their agreement. Reporting both a chance-corrected and a prevalence-robust coefficient at item level, rather than a single pooled statistic, is what made the localization of the disagreement possible.

Several limitations bound the interpretation. The study was conducted in a single tertiary ED with three raters. The observed spread is therefore specific to this setting and these clinicians and should not be read as a generalizable effect size. We would expect the mechanism, rather than its magnitude, to be the more transferable finding, but that expectation is itself a hypothesis these data cannot test. The comparator is the classification of one senior specialist. It was fixed a priori and made independently. It remains a clinical judgment with the same checklist and does not constitute a criterion standard. Agreement with it is not accuracy, and we avoided the vocabulary of diagnostic accuracy for that reason. An independent multi-rater adjudication of the contested cases against written operational definitions would strengthen the comparator and is the natural next step. The checklist itself was not content-validated before use, and no calibration session preceded rating. The between-rater spread should be read with that in mind: it is the reliability of an uncalibrated audit, which is how most audits are run. With only 10 inappropriate catheterizations and 22 cultures, analyses of correlates and microbiological findings were exploratory and underpowered, and multivariable modeling was not appropriate. Ratings were made from ED-period records rather than at the bedside. This reflects how real audits are conducted, but it means that undocumented clinical reasoning was equally unavailable to all three raters. No screening log was kept, so the number of catheterized patients assessed against the eligibility criteria is unknown and the completeness of the consecutive inclusion cannot be quantified; enrollment was continuous and independent of investigator shifts, but we cannot demonstrate that from a denominator. Eligibility was also restricted to patients whose catheter remained in situ for at least 24 h, so catheterizations of a shorter duration, including those removed during the ED stay, are not represented. The reliability estimates reported here therefore apply primarily to this selected population rather than to all ED catheterizations. Day-3 urinalysis and culture were obtained selectively as part of routine care rather than by protocol. The microbiological findings therefore describe a sampled subgroup and cannot be read as incidence. One of the two index raters is an author. We mitigated this by defining the comparator a priori as the independent classification of a non-author senior specialist and by blinding the index raters to one another, though residual bias cannot be excluded. Complications of catheterization were not recorded in structured form and were not analyzed. Finally, the item-level counterfactual analysis is post hoc and hypothesis-generating. It shows that the spread was concentrated in one item in these data, and it identifies a testable prediction, that item-level calibration improves audit reliability, rather than establishing it.

## 5. Conclusions

In this prospective three-rater cohort, one set of 117 ED records yielded an inappropriate-catheterization rate of 0.9%, 8.5% or 25.6% depending on which specialist performed the audit. The 8.5% is the designated senior rater’s. The dependence was not diffuse: two of ten checklist items carried it, and for one item a single rater’s reading accounted for 48% of that rater’s excess endorsements, with replacement of that item’s ratings alone narrowing the spread from 30-fold to 6-fold. In this setting, a single-rater catheter-appropriateness rate did not have the reliability that its use as a quality indicator presupposes. Item-level operational definitions and prospective calibration of auditors should be evaluated as strategies for improving that reliability, and audit reports should give per-item agreement with a prevalence-robust coefficient alongside κ, since the two coefficients answer different questions and their divergence is itself informative. Whether calibration before an audit measurably improves reliability, and how far these findings extend to other departments and other raters, are questions these data motivate but cannot answer.

## Figures and Tables

**Table 1:** Characteristics of the 117 patients undergoing emergency department bladder catheterization.

Characteristic	*n* (%)	95% CI
Age, years, mean ± SD	73.74 ± 13.11	n/a
Age, years, range	21–96	n/a
Sex: women	62 (53.0)	44.0–61.8
Sex: men	55 (47.0)	38.2–56.0
Hypertension	71 (60.7)	51.6–69.1
Diabetes mellitus	39 (33.3)	25.4–42.3
Coronary artery disease	33 (28.2)	20.8–37.0
Heart failure	23 (19.7)	13.5–27.8
Previous cerebrovascular accident	14 (12.0)	7.3–19.1
Chronic kidney disease	9 (7.7)	4.1–14.0
Benign prostatic hyperplasia	1 (0.9)	0.2–4.7
Catheter material: latex	62 (53.0)	44.0–61.8
Catheter material: silicone	55 (47.0)	38.2–56.0
Catheter size: 14 F silicone	8 (6.8)	3.5–12.9
Catheter size: 14 F latex	6 (5.1)	2.4–10.7
Catheter size: 16 F silicone	38 (32.5)	24.7–41.4
Catheter size: 16 F latex	51 (43.6)	34.9–52.6
Catheter size: 18 F silicone	8 (6.8)	3.5–12.9
Catheter size: 18 F latex	4 (3.4)	1.3–8.5
Catheter size: 20 F silicone	1 (0.9)	0.2–4.7
Catheter size: 20 F latex	1 (0.9)	0.2–4.7

Percentages are of the whole cohort (*n* = 117). Confidence intervals are Wilson intervals for a binomial proportion. SD, standard deviation; F, French gauge. n/a, not applicable.

**Table 2:** Inappropriate catheterization under five decision rules applied to the same cohort.

Decision Rule	Patients	Inappropriate, %	95% CI
Index rater 1 alone	1/117	0.9	0.2–4.7
Designated senior rater	10/117	8.5	4.7–15.0
Index rater 2 alone	30/117	25.6	18.6–34.2
Unanimous (all three raters)	1/117	0.9	0.2–4.7
Any rater	30/117	25.6	18.6–34.2

Each row applies a different rule to the same 117 records and the same checklist. Confidence intervals are Wilson intervals.

**Table 3:** Endorsement counts and per-indication agreement for the three raters.

Item	Indication	Senior Rater	Rater 1	Rater 2	Unanimous	Contested	Fleiss’ κ	Gwet’s AC1
1	Acute urinary retention	5	4	0	0	6	0.316	0.964
2	Hourly urine-output monitoring	74	89	68	59	37	0.531	0.617
3	Perioperative use	26	36	31	20	24	0.649	0.776
4	Post-trauma immobilization	8	12	4	4	9	0.597	0.941
5	Neurogenic bladder	6	44	0	0	44	−0.026	0.668
6	Wound care in incontinence	0	1	0	0	1	−0.003	0.994
7	End-of-life comfort	2	0	1	0	3	−0.009	0.983
8	Non-traumatic hematuria	2	1	3	1	2	0.661	0.988
9	Catheter exchange	0	0	0	0	0	NC	1.0
10	No valid indication	10	1	30	1	29	0.199	0.792

Unanimous, patients endorsed by all three raters; contested, patients endorsed by one or two but not all three. NC, not computable: no rater endorsed scheduled catheter exchange, so κ is undefined while AC1 resolves to 1.000. Negative κ values arise from the kappa paradox under skewed marginals.

**Table 4:** Pooled and patient-level agreement between raters, with and without the complementary item.

Comparison	Decisions, *n*	Observed Agreement, %	Positive Agreement, %	Cohen’s or Fleiss’ κ (95% CI)	Gwet’s AC1 (95% CI)
Index rater 1 vs. senior rater, items 1–10	1170	91.2	67.9	0.630 (0.561–0.697)	0.885 (0.859–0.910)
Index rater 2 vs. senior rater, items 1–10	1170	94.0	74.1	0.707 (0.619–0.788)	0.925 (0.904–0.945)
All three raters, items 1–10	1170	n/a	n/a	0.611 (0.551–0.668)	0.886 (0.869–0.903)
Index rater 1 vs. senior rater, items 1–9	1053	91.1	69.7	0.647 (0.580–0.710)	0.881 (0.856–0.905)
Index rater 2 vs. senior rater, items 1–9	1053	95.3	78.3	0.756 (0.678–0.831)	0.941 (0.922–0.958)
All three raters, items 1–9	1053	n/a	n/a	0.652 (0.598–0.707)	0.897 (0.880–0.912)
Index rater 1 vs. senior rater, patient-level verdict	117	92.3	18.2	0.169 (0.000–0.487)	0.916 (0.852–0.965)
Index rater 2 vs. senior rater, patient-level verdict	117	82.9	50.0	0.426 (0.239–0.603)	0.761 (0.640–0.861)
Index rater 1 vs. index rater 2, patient-level verdict	117	75.2	6.5	0.049 (0.000–0.165)	0.678 (0.531–0.794)
All three raters, patient-level verdict	117	n/a	n/a	0.199 (0.060–0.337)	0.792 (0.703–0.864)

Pairwise rows report Cohen’s κ; three-rater rows report Fleiss’ κ. Positive agreement is the proportion of decisions endorsed by both raters among those endorsed by either (for the patient-level verdict, the inappropriate classification). Confidence intervals are percentile bootstrap intervals from 2000 resamples of patients. n/a, not applicable.

**Table 5:** Post hoc counterfactual item-replacement analysis: effect of checklist item 5 (neurogenic bladder dysfunction) on the between-rater range.

Scenario	Index Rater 1, *n* (%)	Senior Rater, *n* (%)	Index Rater 2, *n* (%)	Between-Rater Range
As submitted (all nine valid indications)	1 (0.9)	10 (8.5)	30 (25.6)	30-fold
Item 5 replaced by the senior rater’s ratings for index rater 1	5 (4.3)	10 (8.5)	30 (25.6)	6-fold
Item 5 removed from the checklist for all raters	10 (8.5)	15 (12.8)	30 (25.6)	3-fold

Replacement substitutes the senior rater’s ratings on item 5 for index rater 1’s, leaving all other items unchanged. Removal drops item 5 from the checklist for all three raters, so the senior rater’s estimate also changes. The between-rater range is the ratio of index rater 2’s to index rater 1’s estimate. This analysis is post hoc and hypothesis-generating.

## Data Availability

The data for this study were obtained from the same patient cohort as the residency (medical specialty) thesis of Dr. Ömer Çetin, completed at the Department of Emergency Medicine, Faculty of Medicine, Bolu Abant İzzet Baysal University. Patient identifiers were removed before any analysis, and the dataset was held within the institution throughout. No text, table, or figure is duplicated from either source. The dataset generated and analyzed during the current study is not publicly available because it contains individual-level data whose release would exceed the scope of the participants’ informed consent and of the ethics-committee approval granted for this study (decision no. 2026/10, dated 20 January 2026). De-identified data, together with the analysis script that reproduces every reported value, are available from the corresponding author (farukdanis@ibu.edu.tr) on reasonable request, subject to approval by that ethics committee.

## Supplementary Materials

The following supporting information can be downloaded at: https://www.mdpi.com/article/10.3390/jcm15197574/s1, Table S1: The ten-item catheter-appropriateness checklist as supplied to the raters (English translation); Table S2: Indications for catheterization recorded by the designated senior rater; Table S3: Pooled agreement with the designated senior rater across all 1170 patient-by-indication decisions: observed, positive and negative agreement, chance-corrected coefficients and their gap; Table S4: Generalized linear mixed model of an index rater departing from the designated senior rater’s classification (2340 rater-level assessments); Table S5: Organisms isolated from day-3 urine cultures.

## Abbreviations

The following abbreviations are used in this manuscript:

AC1	first-order agreement coefficient (Gwet’s)
CAUTI	catheter-associated urinary tract infection
CDC	Centers for Disease Control and Prevention
CI	confidence interval
ED	emergency department
GLMM	generalized linear mixed model
HICPAC	Healthcare Infection Control Practices Advisory Committee
OR	odds ratio
SD	standard deviation
STROBE	Strengthening the Reporting of Observational Studies in Epidemiology
