# Supplementary material for: Rater Dependence in Emergency Department Catheter-Appropriateness Audits: A Prospective Three-Rater Study of Item-Level Reliability
Source: J Clin Med. 2026 Sep 29;15(19):7574. doi: 10.3390/jcm15197574 (PMC13665952; doi:10.3390/jcm15197574)
Supplement: Supplementary file 1 [file jcm-15-07574-s001.zip › jcm-4572287-supplementary.pdf]

Supplementary Materials

Table S1. The ten-item catheter-appropriateness checklist as supplied to the raters (English translation).

Item	Indication heading	Descriptor shown to the raters
1	Acute urinary retention / bladder outlet obstruction	Bladder distension detected clinically or by ultrasound (e.g., globe vesicale, prostatic hypertrophy, clot retention)
2	Management of the critically ill patient	Monitoring of urine output in patients with shock, sepsis or polytrauma, or in patients requiring intensive-care follow-up
3	Perioperative use	Urological or genitourinary surgery, or intraoperative fluid-balance monitoring during prolonged operations
4	Post-traumatic immobilization	Restricted mobilization because of spinal or pelvic trauma
5	Neurogenic bladder or neurological dysfunction	Voiding dysfunction after acute spinal injury, stroke, multiple sclerosis or Guillain-Barré syndrome
6	Wound care in the incontinent patient	To prevent urinary contamination in the presence of a sacral or perineal ulcer or burn
7	Palliative / end-of-life care	To provide comfort or drainage at the end of life
8	Non-traumatic hematuria	Drainage where there is a risk of clot retention
9	Exchange / blockage of an existing catheter	Replacement of a previously placed catheter because of blockage or leakage
10	Inappropriate reason (specify)	Convenience, substitute for incontinence management, control during transfer, etc.

The form was administered in Turkish and has been translated into English by the authors; the wording of each descriptor is otherwise unchanged. Each item carried a tick box for present and absent. Items 1 to 4, 6 and 7 follow the appropriate-indications table of the 2009 CDC/HICPAC guideline [3]; items 5, 8 and 9 were added from the Ann Arbor criteria for appropriate urinary catheter use [25]. Decision rule printed on the form: if at least one of items 1 to 9 is ticked, the catheterization is classified as appropriately indicated; if only item 10 is ticked, it is classified as inappropriately indicated. Item 10 is therefore the complement of items 1 to 9. The form was supplied in writing; no calibration session or pilot rating preceded the audit.

Table S2. Indications for catheterization recorded by the designated senior rater.

Indication (checklist item)	Patients, n (%)	95% CI
1. Acute urinary retention or bladder outlet obstruction	5 (4.3)	1.8–9.6
2. Accurate measurement of hourly urine output in critically ill patients	74 (63.2)	54.2–71.4
3. Perioperative use for selected procedures	26 (22.2)	15.6–30.6
4. Prolonged immobilization after potentially unstable injury	8 (6.8)	3.5–12.9
5. Neurogenic bladder dysfunction	6 (5.1)	2.4–10.7
6. Healing of open sacral or perineal wounds in incontinent patients	0 (0.0)	0.0–3.2
7. End-of-life comfort care	2 (1.7)	0.5–6.0
8. Non-traumatic gross hematuria	2 (1.7)	0.5–6.0
9. Scheduled exchange of a chronic indwelling catheter	0 (0.0)	0.0–3.2
10. No valid indication (inappropriate)	10 (8.5)	4.7–15.0

Thirteen patients met more than one valid indication, so the column sums exceed 117 and the percentages exceed 100%. Item 10 is the complement of items 1–9. Confidence intervals are Wilson intervals; for a zero count the interval is one-sided in effect.

Table S3. Pooled agreement with the designated senior rater across all 1170 patient-by-indication decisions: observed, positive and negative agreement, chance-corrected coefficients and their gap.

**Comparison**	**Decisions, n**	**Observed agreement, %**	Positive / negative agreement, %	**κ (95% CI)**	**Gwet's AC1 (95% CI)**	**AC1 − κ**
Index rater 1 vs designated senior rater	1170	91.2	67.9 / 94.9	0.630 (0.561–0.697)	0.885 (0.859–0.910)	0.255
Index rater 2 vs designated senior rater	1170	94.0	74.1 / 96.6	0.707 (0.619–0.788)	0.925 (0.904–0.945)	0.218
All three raters (Fleiss' κ; three-rater AC1)	1170	n/a	n/a	0.611 (0.551–0.668)	0.886 (0.869–0.903)	0.275

Positive agreement is the proportion of decisions endorsed by both raters among those endorsed by either; negative agreement is the corresponding proportion for non-endorsement. Of the decisions the senior rater endorsed, index rater 1 also endorsed 81.95% and index rater 2 75.19%. Confidence intervals were obtained by bootstrap resampling of patients (2000 replicates), preserving the clustering of ten decisions within each patient. κ for the two-rater comparisons is Cohen's κ; for all three raters it is Fleiss' κ. The same coefficients recomputed without item 10, and agreement on the patient-level verdict, are given in Table 4 of the main text.
n/a, not applicable.

Table S4. Generalized linear mixed model of an index rater departing from the designated senior rater's classification (2340 rater-level assessments).

**Term**	**Odds ratio**	**95% CI**	**p-value**
Intercept	0.074	0.056–0.096	<0.001
Age (per year, mean-centered)	0.998	0.984–1.012	0.763
Male sex	0.947	0.651–1.377	0.776

Random intercept for patient identity (standard deviation 0.499). Age is mean-centered; the odds ratio is per year. Confidence intervals are Wald intervals. Reference category for sex is female.

Table S5. Organisms isolated from day-3 urine cultures.

**Organism isolated**	**Isolates, n**	**% of positive cultures**
Enterococcus faecium	3	30.0
Pseudomonas aeruginosa	2	20.0
Candida albicans	1	10.0
Candida glabrata	1	10.0
Enterobacter cloacae	1	10.0
Proteus mirabilis	1	10.0
Escherichia coli	1	10.0

Cultures were obtained in 22 of 117 patients as part of routine care and growth was detected in 10, each yielding a single organism; percentages are of the 10 positive cultures. These findings represent early microbiological growth and were not classified as clinical catheter-associated urinary tract infection.
